# 

**Published:** 1846-05-01

**Authors:** 


BUFFALO

### MEDICAL JOURNAL.

EDITED BY

AUSTIN FLINT, M. D.

**Vol. 1.** **BUFFALO, MAY, 1846.** **No. 12.**

### CONTENTS:

ORIGINAL COMMUNICATIONS.

Notes of an European Tour. No. 9. By F. H. HAMILTON, M. D., Prof. of Surgery in Geneva Medical College. - - - - - - - - - - - - - P. 265
On Turpeth Mineral in Croup. By JOSIAH TROWBRIDGE, M. D. - - - - 270

EDITORIAL, MEDICAL INTELLIGENCE, ETC.

Our Exchange Journals, - - - - - - - - - - - - - - - 271
Treatment of Involuntary Seminal Emissions by Compression, - - - - - - 275
Tertiary Syphilis, - - - - - - - - - - - - - - - - - 276
Citrate of Iron and Sulphate of Quinine in Chlorosis and other Anæmic States, by Dr. Meigs.—Means of arresting Hæmorrhage after excision of Tonsils.—Velpeau's Elements of Operative Surgery, - - - - - - - - - - - - - 277
Professor Paine's Defence of the Medical Profession of the United States, - - - 278
Reports of Lunatic Asylums.—Reports of the Committee on Medical Societies and Colleges of the Senate of New-York, on Lunatic Asylums and the education of Idiots, 279
Senator Backus' Report on Petitions for Homœopathic College.—The Anatomical Remembrancer.—Ranking's Abstract, - - - - - - - - - - - - - 280
Copland's Medical Dictionary.—Braithwaite's Retrospect.—Vaccination after exposure to Small Pox, - - - - - - - - - - - - - - - - - 281
New test for Bile and Sugar, - - - - - - - - - - - - - - - 282
Lectureship on Phrenology,—New Medical School,—Physicians in London,—Acetate of Iron an antidote to Arsenical preparations, - - - - - - - - - - 283
Fragments of Medical Science and Art, an Address by Henry J. Bigelow, M. D., of Boston,—Baltimore College of Dental Surgery,—Ellis' Medical Formulary, Correction,—To our Readers and Correspondents,—Erratum, - - - - - - - 284
Title page and Index to Volume First.
Prospectus of Second Volume of the Medical Journal—Cover, page 2.

BUFFALO:

JEWETT, THOMAS & CO., PRINTERS AND PUBLISHERS,

Exchange Buildings, Third Story, Main-Street.

